# Engineered Tumor-Derived Extracellular Vesicles: Potentials in Cancer Immunotherapy

**DOI:** 10.3389/fimmu.2020.00221

**Published:** 2020-03-06

**Authors:** Adeleh Taghikhani, Farzin Farzaneh, Farzaneh Sharifzad, Soura Mardpour, Marzieh Ebrahimi, Zuhair Mohammad Hassan

**Affiliations:** ^1^Department of Immunology, Faculty of Medical Sciences, Tarbiat Modares University, Tehran, Iran; ^2^Department of Stem Cells and Developmental Biology, Cell Science Research Center, Royan Institute for Stem Cell Biology and Technology, ACECR, Tehran, Iran; ^3^Division of Cancer Studies, Department of Haematological Medicine, King’s College London, London, United Kingdom; ^4^Department of Applied Cell Sciences, Kashan University of Medical Sciences, Kashan, Iran; ^5^Department of Tissue Engineering and Applied Cell Sciences, School of Advanced Technologies in Medicine, Tehran University of Medical Sciences, Tehran, Iran

**Keywords:** cancer immunotherapy, tumor derived exosomes, engineered exosomes, immunomodulatory therapies, immunosuppression

## Abstract

Exosomes are nano vesicles from the larger family named Extracellular Vesicle (EV)s which are released by various cells including tumor cells, mast cells, dendritic cells, B lymphocytes, neurons, adipocytes, endothelial cells, and epithelial cells. They are considerable messengers that can exchange proteins and genetic materials between the cells. Within the past decade, Tumor derived exosomes (TEX) have been emerged as important mediators in cancer initiation, progression and metastasis as well as host immune suppression and drug resistance. Although tumor derived exosomes consist of tumor antigens and several Heat Shock Proteins such as HSP70 and HSP90 to stimulate immune response against tumor cells, they contain inhibitory molecules like Fas ligand (Fas-L), Transforming Growth Factor Beta (TGF-β) and Prostaglandin E2 (PGE2) leading to decrease the cytotoxicity and establish immunosuppressive tumor microenvironment (TME). To bypass this problem and enhance immune response, some macromolecules such as miRNAs, HSPs and activatory ligands have been recognized as potent immune inducers that could be used as anti-tumor agents to construct a nano sized tumor vaccine. Here, we discussed emerging engineered exosomes as a novel therapeutic strategy and considered the associated challenges.

## Graphical Abstract

Imbalance between immune suppression and immune response by tumor derived exosomes. Potential role of engineered tumor derived exosomes in changing the fate of immune response. Image designed by Adeleh Taghikhani.

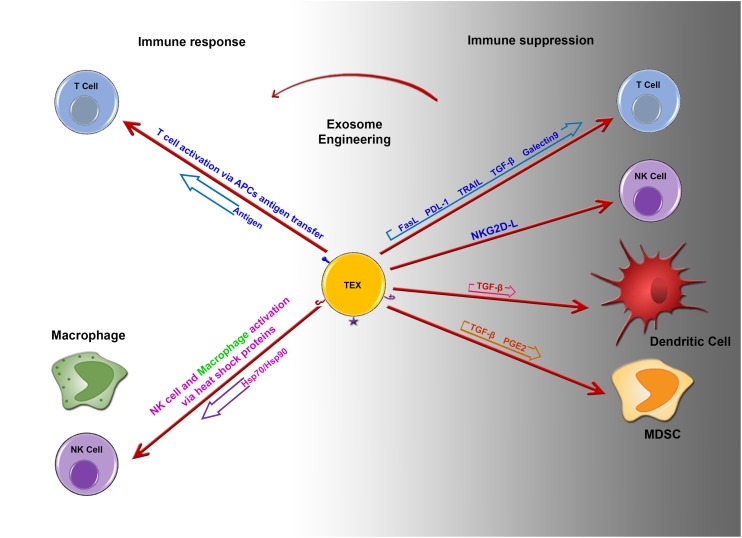

## Introduction

Although cancer immunotherapy has been stablished as a promising treatment option, it faces many challenges including the lack of recognition of specific tumor-associated antigens by the immune system, in part due to the presence of thymic tolerance to self-antigens. Moreover, the cancer microenvironment has immune suppressive properties, thus reducing responses to immune-mediated attacks against the cancer. Tumor microenvironments consist of many cell types that become educated and adapted to support primary tumor growth. Of note, tumor derived exosomes might be involved in this process. In this review, we discuss the role of Tumor derived exosomes in the associated immune suppression and the potential therapeutic applications of modified Tumor derived exosomes, which represents a novel approach for development of therapeutic cancer vaccines.

## Tumor Derived Exosomes (TEXs)

Exosomes are small vesicles (30–120 nm) that are derived from various cells within late endosomes. They are released into the microenvironment where they play a major role in cell-cell communication (1, 2). Exosomes were first considered as waste vesicular bodies of reticulocytes in process of maturation to erythrocytes (3). Moreover, recent experiments support the idea of exosomes could maintain cellular homeostasis (4).

**FIGURE 1:**
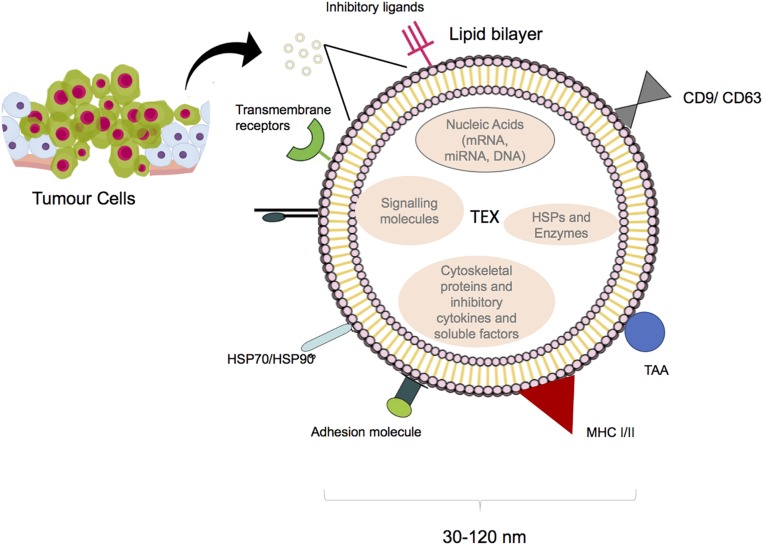
Structure of tumor derived exosome. Size and composition of TEXs. Image designed by Adeleh Taghikhani.

They are generated by inward budding of multivesicular bodies (MVBs) (5), while the exact mechanism of exosome entry into the target cell is not yet clear, phagocytosis and fusion are potentially involved (6, 7). Exosomes are secreted by various cells. For the first time, their release was reported more than 40 years ago as platelets sprinkles (8). Immune cells and cancer cells are also recognized as exosome producers (9–12). Regarding the membrane lipid content, exosomes contain higher concentrations of sphingolipids and cholesterol than that of the cell of origin. They also contain soluble and surface proteins as well as mRNAs and miRNAs. mRNAs result in production of proteins in target cells and miRNAs are transferred between cells and affect expression of different genes (13). Proteomics analysis showed that tumor-derived exosomes (TEXs) contain major histocompatibility complex (MHC) molecules, heat shock proteins, and tetraspanin (CD63, CD81 and CD9) which are known as endosomal pathway proteins. Moreover, tumor antigens such as Mart1, gp100, TRP, and Her2-neu have been found in TEXs (9, 14); (Figure 1). Cancer cells subjected to hypoxic conditions play a role in promoting angiogenesis and metastasis by releasing potent angiogenic factors. They home to metastatic areas through the induction of molecular signals involved in tumor cell recruitment, extracellular matrix deposition, and vascular proliferation. It was also shown that some TEXs contain surface TGF-β along with betaglycan, which could trigger SMAD-dependent signaling and regulate the differentiation of fibroblasts to myofibroblasts (15). In addition, TEXs protect cancer cells from apoptosis through the selective efflux of the apoptosis inducer proteins, which are presented by effector cells such as T cells or natural killer (NK) cells. Moreover, TEXs might diminish the effects of therapy via drug efflux or by masking the binding site of monoclonal antibodies; this could promote the emergence of chemotherapy-resistant cell populations (16).

### Biogenesis of Exosomes

Exosome biogenesis begins with the production of endocytic vesicles via the internalization of surface lipids and clathrin-dependent or independent endocytosis. These vesicles can form early endosomes and late endosomes. Exosomal secretion is achieved by attaching MVBs (microvesicular bodies) to the cell membrane, which appears to be dependent on a variety of Rab GTPase proteins and endosomal sorting complex required for transport (ESCRT). The ESCRT system assists in the sorting of endosomal ubiquitinated proteins for secretion in nanoparticles such as exosomes (17–19).

### Isolation and Characterization

Differential ultracentrifugations have been considered as a gold standard method for effective isolation of extracellular vesicles from conditioned medium which is based on size. In this method, microvesicles, the vesicles which are larger than exosomes, are pelleted at 10,000–20,000 × *g* and the supernatant is subjected to a second centrifugation step at 100,000 × *g*, which results in the isolation of exosomes that are typically smaller than microvesicles.

Other methods for exosome isolation include immunoaffinity purification (affinity chromatography), size exclusion chromatography, polymeric precipitation, microfluidics, and commercial kits based using columns or polymeric precipitations. Exosomes are characterized using a range of techniques including electron microscopy, nanoparticle tracking analysis, dynamic light scattering, flow cytometry and western blotting (20–23). Evidence has shown that the formation of EVs requires function of the endosomal sorting complex required for transport (ESCRT). The typical exosomal protein Alix, which is associated with several ESCRT (TSG101 and CHMP4) proteins, has been reported to participate in endosomal membrane budding as well as exosomal cargo selection therefore these molecules selected as exosomal specific markers in their characterization (24).

## Immunosuppressive Effects of TEXs

Tumor-derived exosomes can carry immunosuppressive molecules such as FasL, TGF-β1, TRAIL, PD-L1, and NKG2D ligands, which are involved in suppression of the immune response (Table 1).

**TABLE 1:** Tumor-derived exosomes involved in suppression of the immune response.

**Immunosuppressive molecule**	**Function**	**References**
Fas-L, placental alkaline phosphatase, B23/nucleophosmin	T cell apoptosis	(28)
Fas-L	CD8 + T cell apoptosis	(25)
Fas-L	Induction of receptor and mitochondrial apoptotic pathways in Jurkat and activated T cells.	(26)
TGF-β	Differentiation to myofibroblast	(15)
TGF-β	T cell suppression	(29)
NKG2D-L	NK cell suppression	(32)
NKG2D-L	NK cell down regulation	(33)
BAG6, BAG4	Tumor evasion	(35)
ND	Treg induction	(39)
ND	Treg induction	(40)
PGE2 and TGF-β	Induction of MDSC expressing Cox2, IL-6, VEGF and arginase-1	(78)
STAT3	MDSC induction	(41)
IL-6	Blockade of DC differentiation	(43)
RIG1 and STAT1	Therapy resistancy	(44)
PGE2	DC dysfunction	(42)
Upregulation of inhibitory genes	Functional decline in T cells	(37)
TGF-β and PD-L1	Drug resistance	(30)

*ND, not defined.*

### Expression of FasL, PD-L1, and TRAIL by TEXs

FasL, TRAIL (TNF-related apoptosis-inducing ligand) and PDL1 (Programmed death-ligand 1) are known as regulator of immune cells homeostasis and activity through inducing of apoptosis following receptor/ligand interactions. The tumor exosome surface may present these regulatory factors to inhibit T-cell proliferation and response through the induction of apoptosis in a dose-dependent manner. This effect can be blocked by the antibody-mediated inhibition of FasL binding to its receptor (25–27). Exosomes isolated from patients with ovarian cancer could express MHC I, placental alkaline phosphatase, TRAIL, and FasL. These exosomes also could suppress the activatory signals of T cells (CD3z and JAK3) following the incubation of TEXs with Jurkat T cells for 48 h (28).

### Expression of TGF-β by TEXs

Transforming growth factor-β1 in exosomes has been proposed to mediate some immunosuppressive effects of TEXs. The presence of TEXs in the monocyte differentiation environment leads to the production of HLA-DR^–/low^ CD14^+^ cells. TGF-β secretion by CD14^+^ HLA-DR^–^ subsets was significantly increased in peripheral blood mononuclear cells isolated from melanoma patients compared to that from healthy subjects (29). It was shown that TEXs might modulate fibroblast phenotype and function via exosomal TGF-β. The mechanism associated with TGF- β exosomal delivery is not limited to SMAD-related pathways, as it was also shown that betaglycans can accompany TGF-β1 on the exosome surfaces, which could facilitate the effects of TGF- β (15). HER2 targeted drug resistance is correlated with TGF-β1 and PDL-1 levels in tumor-derived exosomes. Furthermore, exosomes that carry these molecules could result in adaptation by other drug sensitive cells (30).

### Expression of NKG2D Ligand/BAG6 by TEXs

It was shown that exosomes from murine mammary cell lines can inhibit NK cell cytotoxicity (31). NKG2D is normally expressed on the surface of NK, NKT, activated CD8^+^, and γδ T cells, and its down-regulation in tumor cells is a key mechanism through which cells evade immune surveillance. NKG2D ligands (induced self-proteins) are normally absent on the surface of cells, whereas they are up-regulated in cancers. NKG2D ligands and soluble growth factors including tumor cell secreted TGF-β1 might affect the expression of NKG2D. In NK cells, NKG2D itself is able to trigger cytotoxicity, whereas the function of NKG2D in CD8^+^ T cells is to send co-stimulatory activating signals. In exosome studies, it was found that NKG2D ligands or TGF-β1 on the surface of exosomes can down-regulate NKG2D receptors on effector cells. NKG2D ligands and TGF-β1 expression might affect CD8^+^ T cells and NK cell activity by reducing NKG2D expression on these cells. It is obvious that TEXs harboring a ligand for NKG2D that interacts with lymphocytes do not activate CD8^+^ T cells or NK cells (32).

Blocking ULBP1-5 and MIC A/B in TEXs increases the expression of NKG2D indicating that NKG2D ligand expression in TEXs inhibits NKG2D expression on the surface of NK cells and CD8^+^ T cells, which leads to the suppression of their *in vitro* and *in vivo* loss of function (33, 34). Although, NKG2D ligands on the surface of TEXs were shown to block the activating role NKG2D, one of the NKP30-ligands named BAG6 was expressed on the surface of TEXs and as a soluble molecule; it was sown that the soluble form could promote tumor cell resistance to NK-mediated cytotoxicity, whereas the exosomal form triggered NK cell activation (35).

Although most of experiments have explained the immunosuppressive effects of TEXs on diverse immune cells, they revealed that these structures can provide tumor antigens and heat shock proteins such as HSP70 on their surface which could induce protective anti-tumor immune responses. Gastper et al. 36 suggested that natural killer (NK) cells was stimulated selectively by Hsp70/Bag-4 surface-positive exosomes.

### Induction of Treg Population by TEXs

Tumor derived exosomes can serve as the vehicle responsible for inducing changes in mRNA expression levels in T cells through their miRNA content (37). Human T cells co-incubated with TEXs or exosomes isolated from the plasma of patients with cancer were shown to down-regulate CD3ζ and JAK3 expression in primary activated T cells and mediate the Fas/FasL-mediated apoptosis of activated CD8^+^ T cells. TEXs also promote the proliferation of CD4 + T conventional and their conversion to CD4^+^CD25^high^FOXP3^+^CD39^+^ Tregs, which co-express IL-10 and TGF-β, CTLA-4, and granzyme B/perforin (27, 37) and regulate ADO production by delivering CD73 to the Tregs (38). Thus, TEXs effectively mediate immune suppression. TEXs also increase TGF-β1-associated phospho-SMAD2/3 and phospho-STAT3 levels and IL-10 expression in Tregs (39). T cell response to TEXs is related to surface signaling rather than internalization. Signaling might trigger Ca^2+^ influx or adenosine/A2A R reactions. Recent studies suggest that Tregs are potently induced by these pathways, in contrast to that observed for CD8^+^ or CD4^+^ conventional T cells. This confirms that TEXs could regulate efficient crosstalk between tumor cells and Tregs, which might regulate the tumor environment and immune responses (40). In Tregs, TEXs-mediated down-regulation of genes related to the adenosine pathway results in high expression of CD39 and CD73, as well as increased adenosine production. TEXs also induce the up-regulation of inhibitory genes in CD4^+^ T conv cells, which results in the loss of surface CD69 and a functional decline. Tumor exosomes are not internalized by T cells, but signaling molecules that they carry and deliver to cell surface receptors modulate gene expression and functions in human T lymphocytes. Moreover, TEXs not only induce differentiation and increase expansion of Tregs but also enhance their resistance to apoptosis (39).

### Induction of Myeloid-Derived Suppressor Cell (MDSC) by TEXs

Myeloid-derived suppressor cells have been identified in both human and mouse peripheral blood as a population of immature cells with the ability to suppress T-cell activation. Their accumulation in tumor-bearing mice and human cancer patients was shown to contribute to the development of cancer. Chalmin et al. (41) isolated exosomes from a mouse tumor cell line and demonstrated that the interaction between heat shock protein 72 (HSP72) on the surface of exosomes and the suppressive activity of MDSCs was mediated by the activation of STAT3. In addition, soluble factors derived from tumors increase MDSC induction through Erk pathway activation. HSP72 on the TEXs surface activates STAT3 in MDSCs through TLR2/myd88 and the autocrine production of IL-6. At the molecular level, HSP72 in TEXs, which can act as a ligand of TLR2 in MDSCs, is responsible for the activation of MDSCs and their immune repressive ability. The production of TEXs was decreased using dimethyl amiloride, a drug used to treat high blood pressure, and *in vivo* efficiency of the anti-tumor drug cyclophosphamide was increased in three different mouse tumor models. Overall, the findings in both human and mouse cell lines indicate that HSP72 expression on the surface of TEXs might prevent tumor recognition by the immune system (41).

### Inhibition of DC Differentiation by TEXs

Tumor derived exosomes can target CD11b^+^ myeloid precursors *in vivo*, in mice. Moreover, TEXs prevent the differentiation of mouse myeloid progenitor cells into dendritic cells (DCs) *in vitro*. The addition of TEXs on the 1st day of *in vitro* culture was shown to significantly inhibit the differentiation of monocytes into DCs (42). Similarly, human TEXs inhibit monocyte differentiation *in vitro*. IL-6 and phosphorylated STAT3 levels were increased 12 h after stimulating myeloid precursors cells with TEXs. Moreover, TEXs were less effective in blocking the DC differentiation of monocytes isolated from the bone marrow (BM) of IL-6 knockout mice. The addition of rIL-6 to IL-6 knockout BM cell cultures alleviated this inhibition, restoring DC differentiation. These data indicate that IL-6 from the TEXs plays a major role in blocking the differentiation of BMDCs. Mouse myeloid progenitor cells can take up exosomes and undifferentiated myeloid progenitor cells accumulate in the mouse spleen; subsequently, the differentiation of DCs was found to be inhibited. Although a small number of myeloid progenitor cells can be generated *in vitro* after treatment with myeloid DC exosomes, these cells do not mature and lose their ability to stimulate T-cell activation. Taken together, these results indicate that TEXs-mediated inhibition of DC differentiation might be one of the major mechanisms through which tumor cells evade the immune response, representing a major obstacle for the successful application of immunotherapy to treat cancer (43).

### Other Immunosuppressive Mechanisms of TEXs

Contact between stromal and cancer cells can affect the response to treatment. Boelens et al. (44) showed that the stromal and breast cancer cells utilize paracrine and juxtacrine signaling to acquire resistance to chemotherapy and radiotherapy. The transfer of exosomes from stromal cells to breast cancer cells during their interaction was also demonstrated. RNA species in exosomes, which are largely non-coding elements, stimulate RIG-I receptors and activate STAT1 signaling. In parallel, stromal cells activate NOTCH3 in breast cancer cells. Therefore, stromal cells use exosomes to stimulate and orchestrate signaling in breast cancer cell subpopulations to acquire resistance to treatment and mediate tumor recurrence (44).

Although these findings explain the immunosuppressive effects of TEXs on diverse immune cells, they also suggest that these structures can provide tumor antigens for antigen processing cells and enhance the chances of tumor antigen recognition by immune cells. Dendritic cells pulsed with TEXs can prime cytotoxic T cells to induce protective anti-tumor immune responses. Furthermore, high levels of Hsp70 in TEXs were reported to elicit a direct Th1-polarized immune response. In addition, NK and NKT activation by TEXs was also reported (36, 45).

## Strategies for Increasing Immunostimulatory Effects of TEXs

According to the aforementioned results, it seems that appropriate responses from the host against TEXs will only be induced by engineering them; accordingly, several studies have been conducted and their results are proof of this claim. Here, we discuss strategies devised over the past few years to circumvent this problem, immunosuppressive properties of TEXs, along with their possible applications and limitations.

### Genetic Engineering

Tumor derived exosomes could be manipulated by tumor-associated genes or some known immune boosters such as CpG DNA and TLR ligands and overexpress them to induce cellular and innate immunity; they could also be targeted using genetic materials such as siRNA, which can be introduced by electroporation into purified exosomes. Using this approach, nerve cells were previously targeted and gene silencing was confirmed (46). As a novel vaccine for cancer therapy, exosomes secreted by B16 melanoma cells modified to express both tumor-associated antigens and the pathogenic antigen, which increased cellular immunity against *Mycobacterium tuberculosis* and resulted in suppression of tumor growth in tumor-bearing mice (47). Moreover, streptavidin and lactadherin expressing exosomes (SAV-exos) used in combination with biotinylated CpG DNA and immunization with CpG-SAV-exos resulted in strong anti-tumor effects in tumor-bearing mice (48).

### miRNA Modification

Exosomes secreted from cell lines that were treated with DHA (docosahexaenoic acid) were shown to express higher levels of let-7a, miR-23b, miR-27a/b, miR-21, let-7, and miR-320b, which are tumor suppressor miRNAs. Further untreated epithelial cells incubated with the exosomes are also shown to have increased levels of those miRNAs. The data showed that DHA, as an anti-angiogenic factor, can affect miRNA levels in tumor exosomes. Therefore, we could target exosome signaling between tumor cells in the cancer microenvironment (49). Also, we found that Let-7i and miR-142 upregulation in TEX have notably enhancing effect on either DC maturation or CTL induction and cytokine release (50).

Moreover, ultra-filtered exosome lysates (UELs), which can be prepared for the depletion of miRNAs from exosomes, were assayed and the resulting protein extraction was used for dendritic cell activation. The results indicate that miRNA-depleted TEXs proteins might be promising agonists for the specific activation of DCs (51).

### Conjugation

The binding of some molecules triggers immune cells to naturally become potent effectors. Furthermore, the binding of such activator ligands or co-stimulators to TEXs might increase their effectiveness in difference processes such as attaching to TLR ligands like CpG-DNA, Poly:IC and IL15Rα or activation of NK cells by using NKG2D ligands. Since, exosomes produced by immature monocyte derived DC naturally present Bat3 and nkp30 ligands on their surface, leading to diminished NK cell responses (52).

In addition, we should mention another opportunity; VAR2CSA is a malarial protein that can bind the VAR2CSA-ligand or chondroitin sulfate (CS) expressed in the placenta. CS is also present on a high proportion of malignant cells and could be targeted by recombinant VAR2CSA (rVAR2). Salanti showed that this rVAR2 could bind placental-type CS as well as tumor cells (PC-3, MDA-MB-231, and MyLa2059). They proposed that targeting the common CS chain, which is present on different cancer cells, might offer a novel cancer treatment strategy (53, 54) furthermore, VAR2CSA upregulated TEXs could be the next level of this vaccination procedure.

The use of exosomes for protein loading via optically reversible protein–protein interactions (EXPLORs) is a new strategy that was described for the intracellular delivery of target proteins. This occurs through the integration of a protein–protein interaction module that is controlled by blue light during endogenous exosome biogenesis (55). It has also been shown that the IFN gamma and its target gene, IRF-l has important role in tumor cell apoptosis. TEXs released from IRF-1 primed cells containing increased level of IL-15R and MHC-I and could promote antitumor immunity by CD4 + and CD8 + T cells infiltration (56).

### HSP70/90 Overexpression (Heat Treatment)

It has been shown that exosomes isolated from tumor cells express heat shock proteins including the cognate 71-kDa HSP, the 70-kDa HSP4, and HSP90 alpha and beta on their surfaces (57). Moreover, tumor-derived chaperon rich cell lysates containing Hsp70, Hsp90, calreticulin, and glucose-regulated protein 94 have been reported to activate DCs (58). Bu et al. (59) demonstrated that exosomes from DCs loaded with chaperone-rich cell lysates could elicit a potent T cell immune response against intracranial glioma in mice. These findings suggested that chaperons are potent candidates to increase the immunostimulatory effect of TEXs.

### Lipid Modification and Glycosylation

Hybrid exosomes, which are developed by fusing their membranes with liposomes (60–62), can be combined with genetic modification techniques. The interactions between the engineered exosomes and cells could be modified by changing the lipid composition or the properties of the exogenous lipids, which was shown to facilitate drug delivery or antigen presentation to immune cells located in the lymph node (62) and delivered CRISPR/Cas9 system in Mesenchymal Stem cells (MSCs) to alleviate *in vivo* gene editing (63). Glycosylation was identified as a promising strategy for stabilizing peptides on the surface of exosomes; this was shown to protect them from proteolytic degradation and effectively direct exosomes to specific destinations *in vivo*. This strategy could enhance the display of targeting peptides and generally enable and improve exosome-based therapeutics (64).

### Future Vesicle-Based Cancer Therapies

As drug delivery vehicles, exosomes could be used for carrying various hemotherapeutic agents. Curcumin (natural polyphenol compound) which its anti-inflammatory effect have been shown in several studies (65–67) was packed in exosomes to enhance curcumin effectiveness (68, 69). Paclitaxel, doxorubicin, and temozolomide could be packed into exosomes. siRNAs or anti-miRNA oligonucleotides might also be used as therapeutic cargos carried by exosomes (70). Moreover, novel technologies like nanoparticles (cationic liposomes) containing tumor RNAs, known as RNA lipoplexes (RNA-LPX), have been established. These RNA-LPXs were shown to induce immune responses (71–74). An *in vivo* study using intravenous vaccination demonstrated the homing of RNA-LPXs to lymphoid tissues and stimulation of macrophages and DCs, which promoted T cell responses via IFN-α release and Th1 and CD8^+^ polarization (72). MSCs are particularly interesting as they localize and migrate toward damaged and inflammatory microenvironments including solid tumors, however they display multi-functional activities with both pro- and anti-tumor effects within the microenvironment; thus, MSC-derived exosomes could represent a potential anti-tumor vaccine (75) or drug delivery strategy (76, 77).

## Conclusion

As discussed, TEXs play a major role in tumor immune evasion and growth. In addition, they protect tumor cells from chemotherapy and immunotherapy via the efflux the chemicals and the masking of monoclonal antibody binding sites. TEXs, comprising a critical part of the tumor microenvironment, have been proposed as markers for the early diagnosis of cancers. Although evidences suggest that we should target TEXs for cancer treatment, it might be time to address this from a different point view. Based on data that we described herein, we suggest that tumor derived extracellular vesicles could be manipulated and provide cancer treatment. In this way, tumor-associated antigens, unrecognized antigens in the cytosol of TEXs, and other molecules such as HSPs could be exploited in the future for cancer treatment.

## Author Contributions

All authors have contributed to organizing the main idea. ME and ZH helped, supported, and supervised. AT developed the idea and wrote the manuscript. FF, FS, and SM contributed in editing.

## Conflict of Interest

The authors declare that the research was conducted in the absence of any commercial or financial relationships that could be construed as a potential conflict of interest.
